# Study on Fractal Characteristics of Mineral Particles in Undisturbed Loess and Lime-Treated Loess

**DOI:** 10.3390/ma14216549

**Published:** 2021-11-01

**Authors:** Jian Song, Jiaxin Ma, Fengyan Li, Lina Chai, Wenfu Chen, Shi Dong, Xiaojun Li

**Affiliations:** 1 Shaanxi Provincial Communication Investment Group Co., LTD, Xi’an 710065, China; songsong163163@126.com; 2 College of Safety Science and Engineering, Xi’an University of Science and Technology, Xi’an 710054, China; m18309254342@126.com (J.M.); lfy180104@163.com (F.L.); CLN12162021@163.com (L.C.); 3 Anhui Water Conservancy Development Co., LTD, Bengbu 233000, China; wenfuchen163@163.com; 4 College of Transportation Engineering, Chang’an University, Xi’an 710064, China; dongshi@chd.edu.cn

**Keywords:** highway subgrade, loess, lime-treated loess, mineral particles, multifractal, non-uniformity

## Abstract

In order to explore the fractal characteristics of particle size distribution (PSD) of various minerals in loess and lime-treated loess, the Q4 undisturbed loess and lime-treated loess were studied. From the perspective of multi-scaled microstructure, the internal characteristics of loess were observed and the regularity statistics were carried out from a macroscopic view. Fractal theory was used to quantitatively study the distribution of mineral particles in undisturbed loess and lime-treated loess. It was found that the skeleton particles of undisturbed loess were obvious and the structure of soil was loose. While that of lime-treated loess decreased, the fine particles were connected with each other, and the structure of soil changed from loose to dense. The three mineral particles in the undisturbed loess and lime-treated loess did not accord with the single fractal distribution characteristics, but the total particles had fractal characteristics. The percentage content of the mineral particles in the soil varied greatly with the particle size. In addition, the non-uniform degrees of mineral particles in the two soils from large to small were carbonate minerals of lime-treated loess, carbonate minerals of undisturbed loess, quartz minerals of lime-treated loess, feldspar mineral of lime-treated loess, feldspar mineral of the undisturbed loess, and the quartz mineral of the undisturbed loess. This paper provided a basis for the future study of the different soil mechanical properties of undisturbed loess and lime-treated loess.

## 1. Introduction

Loess is a widely-distributed typical structural soil [1,2,3]. Therefore, it is used as a common building material in highway subgrade or roadbed. However, it is a complex porous medium composed of different mineral particles with irregular shapes. At the same time, its macro complexity features such as discontinuity, non-uniformity, and anisotropy are closely related to its microstructure [4]. Therefore, these characteristics cause certain challenges for highway engineering in the loess area.

In recent years, important achievements have been made in the study of soil structure by using fractal and multifractal theory, from the micro scale (μm) to the macro scale (km) [5,6]. The establishment and application of fractal theory provide a new approach to quantitatively describe the particle size distribution (PSD) of soil mineral particles. Fractal dimension can characterize the difference and self-similarity of soil particle distribution, and reflect its uniformity [7]. Xie et al. systematically studied the pore fractal and particle fractal of rock and soil materials, and proposed the measurement method of pore fractal [8]. Liu et al. pointed out that the particle size fractal dimension could reflect the pore and microstructure characteristics of soil, and it was a method of characterizing the non-uniformity of cohesive soil [9]. Hu Ruilin and Zhang Jiru measured the equivalent diameter and distribution of soil particles by Housdoff fractal dimension calculation method and computer image analysis technology [10,11]. The quantitative results of particle distribution fractal dimension, surface characteristic fractal dimension, and pore and contact zone distribution fractal dimension were obtained. The fractal dimension of soil was characterized by the quantitative distribution of particle size, which provided a method for the study of fractal structure. The fractal dimension of soil particles, aggregates and porosity is calculated using the single fractal theory to characterize the composition and uniformity of soil structure, which provides an accurate and simple method for quantitative description of soil structure characteristics. In addition, at present, many scholars use multifractal theory to study the characteristics of soil particle size distribution, and multifractal method can reflect the local heterogeneity and non-uniformity of soil structure in detail [12]. Grout et al. and Posadas et al. believe that the particle size distribution of soil particles does not conform to the principle of single fractal, and the multifractal method can more accurately analyze the PSD characteristics [13,14]. Dong et al. found that the capacity dimension D(0) and information dimension D(1) could be used as potential indicators to reflect the physical properties and mass of soil [15]. Guan et al. concluded that the multifractal spectrum parameters of soil particle size distribution can reflect the non-uniformity of soil particle size distribution [16].

However, due to the limitation of test technology, most of these studies only analyzed the fractal characteristics of the overall particle size distribution of soil, while fewer studies focused on the fractal characteristics of different mineral particles under different soils. The SEM-EDX study was widely used in cement and concrete research [17,18,19]. In this paper, the multi-scale microstructure images obtained by image scanning, processing, and mineral composition identification were more representative and could better reflect the characteristics of macroscopic soil samples. The fractal characteristics of each mineral particle in Q_4_ undisturbed loess and lime-treated loess were quantitatively analyzed, and the distribution characteristics of single fractal and multi-fractal of three mineral particles in the two soils were discussed, thus providing a basis for studying different soil mechanical properties of natural loess and lime-treated loess.

## 2. Materials and Methods

### 2.1. Sampling

The Q_4_ undisturbed loess sample was taken from the exploratory well of Xining Qinghai-Tibet Science and Technology Museum, which is located in Xining City, Qinghai Province. The untreated loess sample, 6~8 mm in height, was cut from the original ring knife sample and was pressed into the soil extractor. The main physical properties are as follows: natural water content *w* is 16.7%, soil specific gravity G_s_ is 2.70, natural bulk density r is 16.3 kN/m^3^, void ratio *e* is 0.93, dry density ρ_d_ is 1.4 g/cm^3^, liquid limit is 24.8%, and the plastic limit is 15.4%. A part of the soil samples was crushed, air-dried, and sieved through an aperture of 2 mm. Lime was weighed, and according to a mass ratio of 100:7, mixed evenly with loess to prepare the lime-treated soil samples with water content of 16.7% and dry density of 1.39 g/cm^3^. It was pressed into the soil extractor for sample preparation, drying, curing, coarse grinding, fine grinding, polishing until the surface of the soil sample showed flatness and smoothness, which can be observed by electron microscope scanning, and then it was coated with gold sprayer for further use.

### 2.2. Sample Image Processing

**Figure 1:**
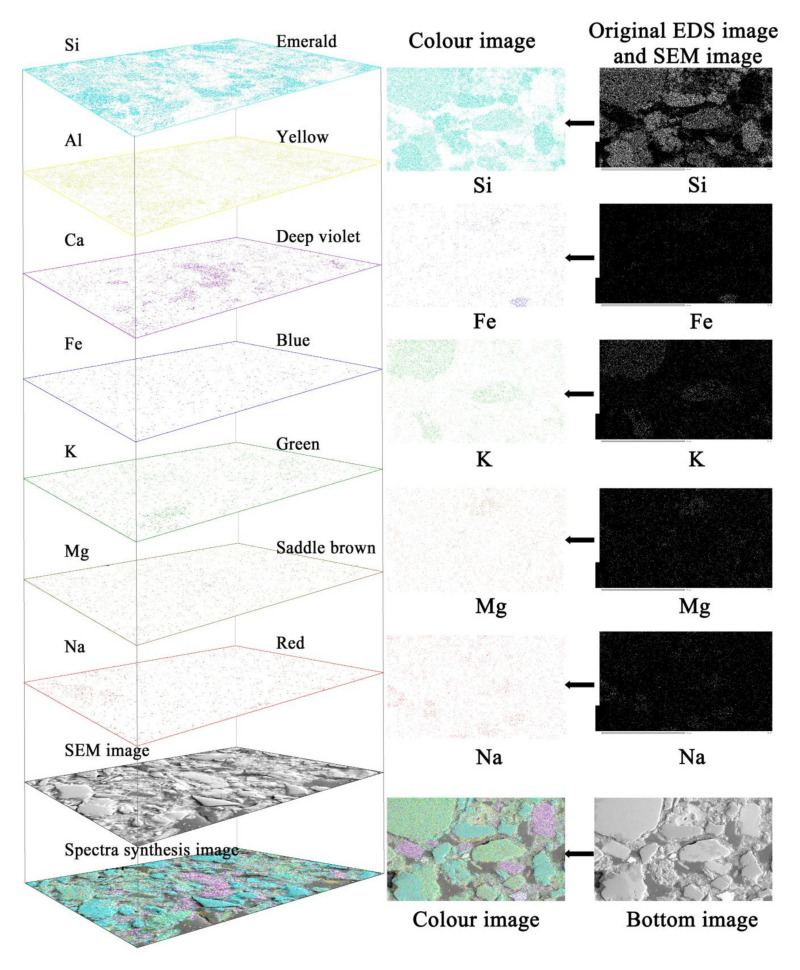
Element overlay schematic.

The sample was scanned with a JSM-6390A scanning electron microscope at a magnification of 500 times. After scanning, an energy spectrum base map and a surface scanning image of seven elements Si, Al, Ca, K, Fe, Mg, and Na were obtained; 320 images (including 40 base map and 280 EDS photo for 7 different elements) were obtained for each sample (Figure 1). Corresponding colors fell respectively into seven different elements, and then these seven different colors were added to the base map according to the RGB values [20], as shown in Figure 1.

**Figure 2:**
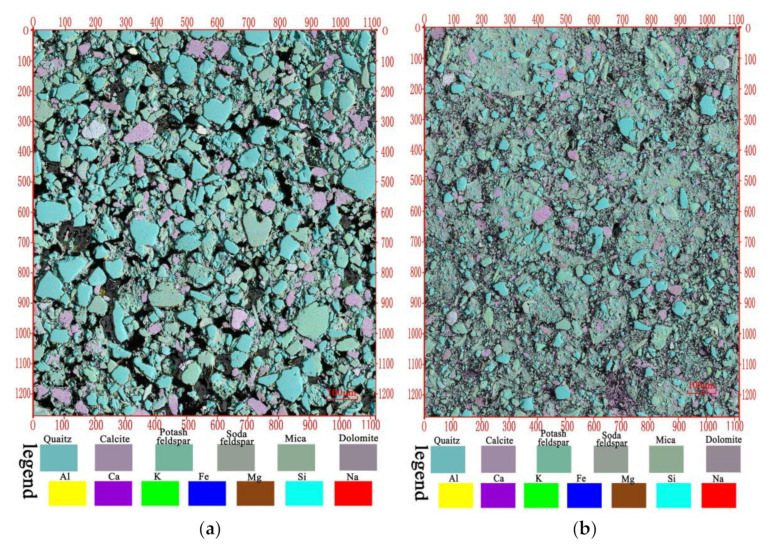
Multi-scale microstructure of undisturbed loess and lime-treated loess from Xining. (**a**) Undisturbed loess; (**b**) Lime-treated loess.

In order to ensure complete and seamless splicing, the images were spliced in the form of an S-shaped route with a horizontal overlapping width of 1/4 and a vertical overlapping length of 1/3. The multi-scale microscopic image of mineral composition distribution in the two soils was obtained and transformed from micron to millimeter scale, as is shown in Figure 2.

**Figure 3:**
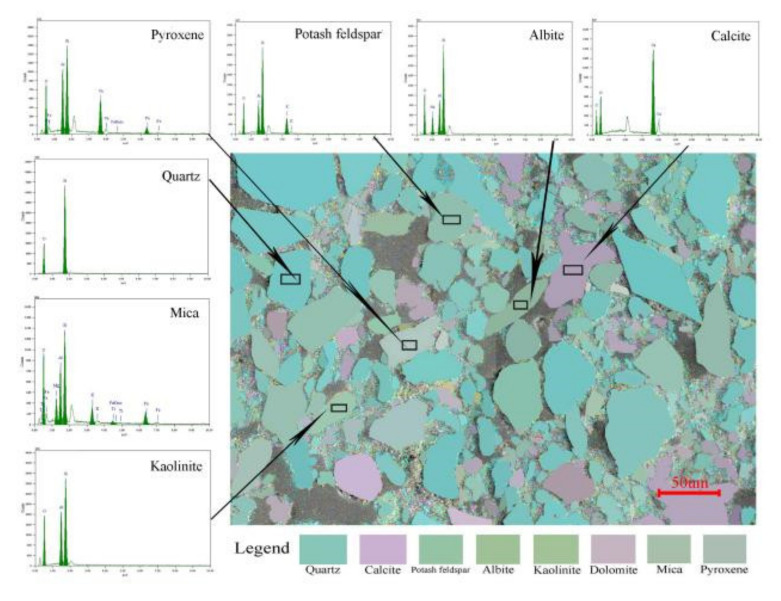
Standard database validation schematic.

After the microscopic image was processed, the chromatic value (a1, b1) of unknown minerals was obtained by averaging. According to the formula ΔE = ((Δa)2 + (Δb)2)1/2, the difference between the chromaticity value of unknown minerals and the standard chromaticity value of minerals was calculated. Δa = a − a1, Δb = b − b1, where (a,b) is the standard color of mineral A. If ΔE < 1, the mineral was identified as A. The identification results of mineral image recognition were compared with those of energy spectrum to verify the accuracy of the method. The validation schematic is shown in Figure 3. All the minerals in the schematic have energy spectrum data, and the mineral name can be identified.

Based on this method, quartz, feldspar, and carbonate mineral particles in untreated loess and lime-treated loess were identified respectively, and the particle size parameters of each mineral particle in the two soils were extracted.

### 2.3. Distribution Characteristics of Mineral Particle Size

It can be observed from Figure 2 that the skeleton particles of undisturbed loess are obvious and the particles support each other to form a macroporous support. The skeleton particles were clearly distinguished from the pores, and the particle contour was clear. While the skeleton particles of lime-treated loess decreased, the fine particles were connected with each other, and many small particles were closely combined with clay minerals to form new aggregates with large particle size. These new aggregates were composed of massive, rounded small quartz, carbonate, and clay minerals, etc., with particle sizes ranging between 5 and 20 μm.

**Figure 4:**
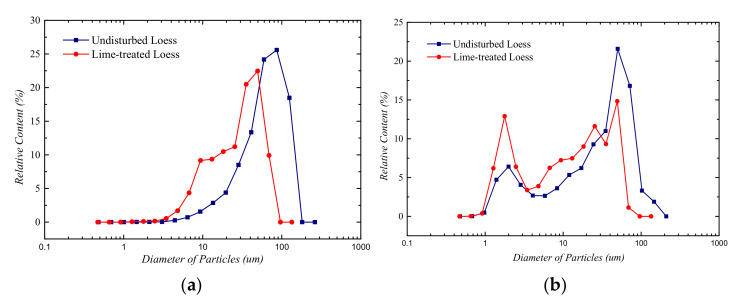

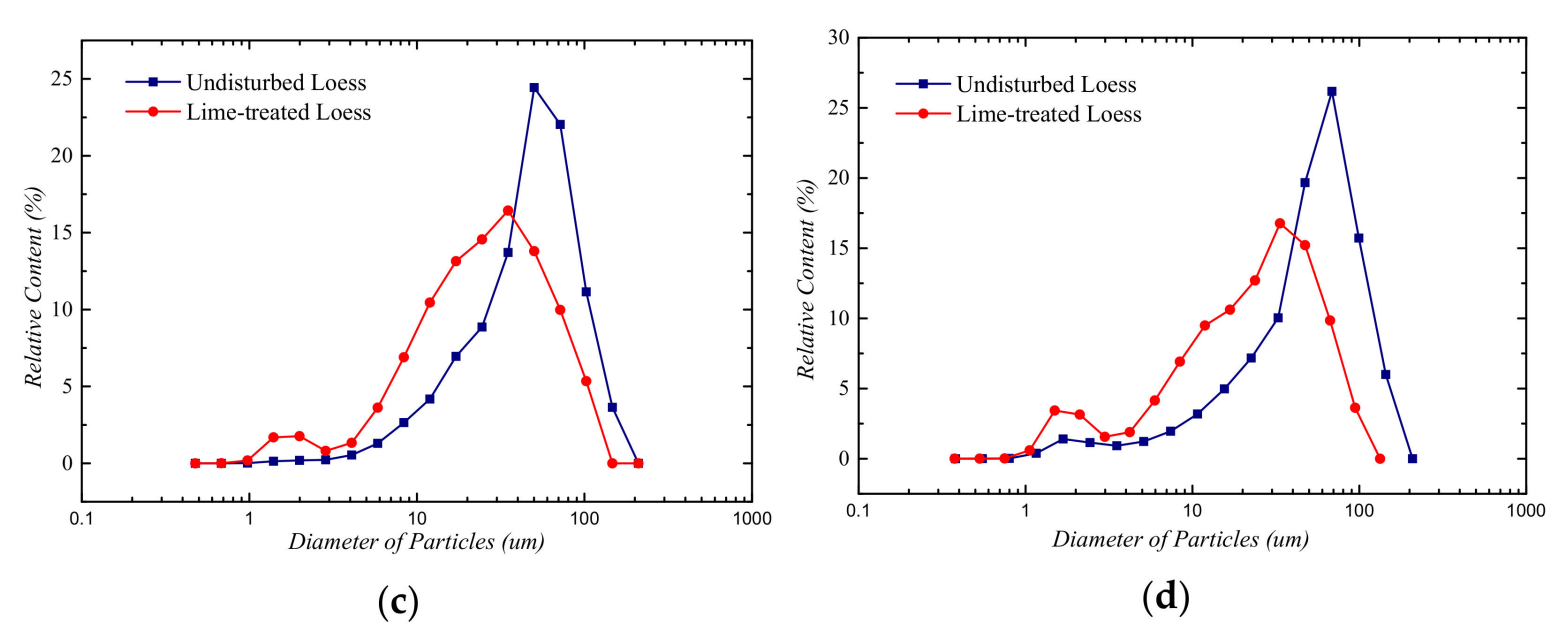
Particle size distribution curves of minerals in undisturbed loess and lime-treated loess. (**a**) Particle size distribution curve of quartz minerals; (**b**) particle size distribution curve of carbonate minerals; (**c**) particle size distribution curve of feldspar minerals; (**d**) particle size distribution curve of total minerals.

The particle size parameters of each mineral particle in natural loess and lime-treated loess were extracted, and the particle size interval I = [0.02,2000] was selected according to the logarithmic equidistant method. The distribution of different mineral particle sizes was calculated, and the spatial distribution characteristics were analyzed and compared. The particle size distribution curves of various minerals in the two soils are shown in Figure 4.

It can be seen from Figure 4a that in the range of particle size 1~10 μm, the quartz content in lime-treated loess was about 10%, and that in undisturbed loess was about 2%. In the range of 10~100 μm, the content of quartz particles in undisturbed loess was 27% at the particle size of 100 μm, whereas the corresponding particle size of quartz particles in lime-treated loess was about 40 μm and the content was about 23%. In Figure 4b, there are two peaks in the particle size distribution curve of carbonate minerals in undisturbed loess, the corresponding particle sizes are 2 μm and 70 μm, and the contents are about 7% and 22%, respectively. There were three peaks in the distribution curve of carbonate minerals in lime-treated loess, and the corresponding particle sizes were 2 μm, 27 μm, and 50 μm, respectively. The percentages of particle content were 13%, 12%, and 15%, respectively. From the particle size distribution curve, the carbonate mineral particle size distribution of lime-treated loess was not uniform. Figure 4c shows that the main particle size of feldspar minerals in undisturbed loess was 60 μm and the content was about 26%. There are two peaks of feldspar minerals in lime-treated loess, the corresponding particle sizes were 2 μm and 70 μm, and the contents were 2% and 16%, respectively. Compared with undisturbed loess, the particle size of feldspar minerals in lime-treated loess decreased and the particle size distribution was non-uniform.

From the whole mineral particle size distribution curves, compared with undisturbed loess, the particle size of all mineral particles in lime-treated loess decreased. This is consistent with the decrease of mineral particles in lime-treated loess observed directly above.

## 3. Fractal Theory

### 3.1. Single Multifractal Calculation

Soil is a complex porous medium with fractal characteristics, and the fractal can be defined by the relationship between particle size and number of soil particles [21].

(1) $$N(\delta >d_{i})=Cd_{i}{}^{-D}$$

where, $N(\delta >d_{i})$ is the total number of particles, which is larger than $d_{i}$. C is a constant related to soil properties. D is a fractal dimension.

Assuming that the total number of soil mineral particles is $N_{T}$ and $d_{\min}$ is the minimum particle size of mineral particles, it can be obtained from Equation (1).

(2) $$\frac{N_{T}}{N(\delta >d_{i})}={\left(\frac{d_{i}}{d_{\min}}\right)}^{D}$$

The slope k of the straight line is obtained through linear fitter with $\log(d_{i}/d_{\min})$ and $\log(N(\delta >d_{i}))$ as abscissa and ordinate respectively. If these points satisfy the linear relationship, the fractal dimension of mineral particle size distribution D=k, thus showing the mineral particles in undisturbed loess and lime- treated loess of Xining Q_4_ have single fractal features.

### 3.2. Multifractal Calculation

A dimensionless interval J=[0,5] [14,15,16] is obtained by logarithmic transformation based on the particle size interval I=[0.02,2000]. In the interval J, there are $N(\varepsilon )=2^{k}$ subintervals with size $\varepsilon =5\times 2^{-k}$, and k is 1 to 6. Construct a family of partition functions using $p_{i}(\varepsilon )$ is shown as Equation (3) [22].

(3) $$u_{i}(q,\varepsilon )=\frac{p_{i}{\left(\varepsilon \right)}^{q}}{\sum_{i=1}^{N(\varepsilon )}p_{i}{\left(\varepsilon \right)}^{q}}$$

where, $u_{i}(q,\varepsilon )$ is the q-order probability of the *i* subinterval, q is a real number, $\sum_{i=1}^{N(\varepsilon )}p_{i}{\left(\varepsilon \right)}^{q}$ is the sum of q-order probabilities of all subintervals.

Then the multifractal generalized dimension spectrum D(q) is calculated as Equation (4) [20].

(4) $$D_{q}=\underset{\varepsilon \to 0}{\lim}\frac{1}{q-1}\frac{\mathrm{lg}(\sum_{i=1}^{N(\varepsilon )}p_{i}{\left(\varepsilon \right)}^{q})}{\mathrm{lg}\varepsilon }$$

When q=0, D(0) is the capacity dimension; when q=1, D(1) is the information dimension; when q=2, D(2) is the correlation dimension.

Singularity index of mineral particle size distribution α(q) can be calculated as Equation (5) [23].

(5) $$\alpha (q)=\underset{\varepsilon \to 0}{\lim}\frac{\sum_{i=1}^{N(\varepsilon )}u_{i}(q,\varepsilon )\log u_{i}(\varepsilon )}{\log(\varepsilon )}$$

The multifractal spectrum function f(α) can be calculated as Equation (6):

(6) $$f(\alpha )=\underset{\varepsilon \to 0}{\lim}\frac{\sum_{i=1}^{N(\varepsilon )}u_{i}(q,\varepsilon )\log u_{i}(q,\varepsilon )}{\log(\varepsilon )}$$

In the range of −10≤q≤10, fitting with 1 as step length, the generalized dimension spectrum (D(q)), singularity index (α(q)), and multifractal spectrum function ((f(q)) of mineral particle size distribution in undisturbed loess and lime-treated loess are calculated using Equation (3) to (6).

## 4. Results and Analysis

### 4.1. Mineral Particle Size Comparison

It can be speculated that in the process of crushing and compaction, large particles are crushed to many small particles and a series of chemical and physicochemical reactions occur between lime and soil to give rise to cements, such as calcium carbonate and crystal calcium hydroxide, etc. The aggregation of clay colloid particles makes the soil structure dense.

From the whole mineral particle size distribution curves, compared with undisturbed loess, the particle size of all mineral particles in lime-treated loess decreases. This is consistent with the decrease of mineral particles in lime-treated loess observed directly above. In terms of the non-uniformity of mineral particle distribution, the carbonate mineral particle distribution in lime-treated loess is the most uneven. This is the result of a series of chemical and physicochemical reactions in the soil after lime is added, which gives rise to cements, such as calcium carbonate and crystal calcium hydroxide.

### 4.2. Single Fractal of Mineral Particle Distribution

**Figure 5:**
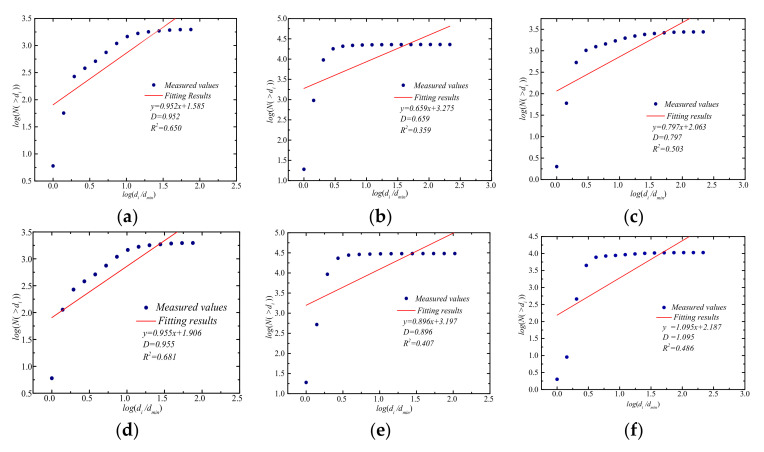
Fractal dimension of the number of mineral particles in undisturbed loess and lime-treated loess. (**a**) Quartz minerals in undisturbed loess; (**b**) carbonate minerals in undisturbed loess; (**c**) feldspar minerals in undisturbed loess; (**d**) quartz minerals in lime-treated loess; (**e**) carbonate minerals in lime-treated loess; (**f**) feldspar minerals in lime-treated loess.

According to Equation (2) and the PSD curves of each mineral particle in natural loess and lime-treated loess, $\log(d_{i}/d_{\min})$ and $\log(N(\delta >d_{i}))$ curves can be plotted, and the fractal dimension of particle size distribution of each mineral particle can be obtained, as shown in Figure 5.

For different properties of soil, the fractal dimension of particle size distribution reflects the particle size and distribution uniformity. The larger the fractal dimension is, the smaller the particle size of soil mineral particles is, the higher the fine particle content is, and the more uneven the texture is. The fractal dimension in equation 2 reflects the characteristics of particle size distribution of soil mineral particles, and has a clear physical meaning. It shows that when D=0, the soil is completely composed of mineral particles with equal particle size. Most fractal dimensions of soil mineral particles determined by particle size distribution are between 1.0 and 3.0, and some are greater than 3.0 [11]. Since the mineral particle data in this paper were obtained according to the parameters such as particle area and equivalent diameter, etc., the fractal dimension ranged between 1 and 2.

It can be seen from Figure 5 that the fractal dimensions of quartz, carbonate, and feldspar mineral particles in undisturbed loess were 0.952, 0.659, and 0.797, respectively; the fractal dimensions of quartz, carbonate and feldspar mineral particles in lime-treated loess were 0.955, 0.896, and 1.095, respectively. Most of the fractal dimensions ranged from 0 to 1.0, which does not conform to the range of particle size fractal dimension, and the fitting accuracy is in the range of 0.359~0.681, which is not satisfying. It shows that the distribution of mineral particles in the two soils does not accord with the characteristics of single fractal.

It can be seen from the particle size distribution curve of mineral particles in Figure 4 that the smaller the curve’s amplitude of variation was, the greater the non-uniformity of mineral particle distribution was, which indicates the mineral particle content in each particle size tended to be consistent. The mineral particle content of different particle size in the two soils was unevenly distributed, whereas the particle size distribution of carbonate minerals showed great non-uniformity, which also indicates that single fractal can only describe the overall characteristics of particle distribution rather than the local characteristics of soil structure. Therefore, it is possible to analyze the distribution of mineral particle size by multifractal theory, which can reflect the local heterogeneity and non-uniformity of the distribution of mineral particles in more detail.

### 4.3. Multifractal of Mineral Particle Distribution

#### 4.3.1. Generalized Dimension Spectrum Curve q−D(q)

According to the multifractal theory, when D(0) is larger, the range of mineral particle size distribution is wider; when D(1) is larger, the distribution range of soil mineral particles is wider, and the percentage of mineral particle content in each region is evenly distributed at various scales. The value of D(1)/D(0) can reflect the dispersion degree of particle size distribution. If D(0)=D(1)=D(2), the distribution of soil mineral particles has a single fractal structure. The values of D(0), D(1), D(1)/D(0) of mineral particles in undisturbed loess and lime-treated loess are shown in Table 1.

As is shown in Table 1, D(0)>D(1)>D(2) applies in all mineral particles—quartz, feldspar and carbonate in untreated loess as well as in lime-treated loess, indicating that the particle size distribution of the three minerals in the two soil samples is non-uniform fractal, which also shows that it is necessary and reasonable to analyze the PSD of each mineral in undisturbed loess as well as lime-treated loess by the multifractal method.

**Figure 6:**
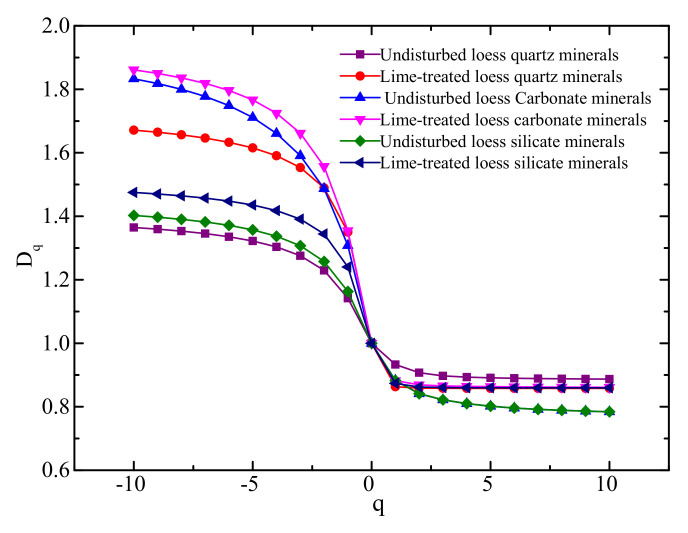
Generalized dimension spectrum curve q−D(q) of mineral particles in undisturbed loess and lime-treated loess.

On the basis of the multifractal analysis of three kinds of mineral particles in undisturbed loess and lime-treated loess, the generalized dimension spectrum curve q−D(q) of PSD of mineral particles is obtained in the range of -10≤q≤10, as shown in Figure 6.

For non-uniform fractal, q−D(q) had a certain width, and the greater the curvature was, the worse the soil uniformity was [24]. PSDs of the three mineral particles had a certain degree of curvature and showed a certain degree of non-uniformity, and the carbonate mineral particles in lime-treated loess were the most obvious.

Figure 6 shows that with the increase of q, the D(q) of the three mineral particles in two soil samples decreased, and when q>1, the decreasing trend slowed down, and the generalized fractal dimensions of different mineral particles approached 0.8; D(q) of carbonate minerals in undisturbed and lime-treated loess changed most obviously with the increase of q, showing stronger non-uniformity than PSD of other minerals; D(q) of quartz minerals in undisturbed loess changed the least with the increase of q, indicating that its PSD was relatively uniform; D(q) of quartz and feldspar mineral particles in lime-treated loess increased with q, which is larger than that of quartz and feldspar mineral particles in undisturbed loess, but far less than that of carbonate mineral particles, showing moderate particle size distribution characteristics of mineral particles.

#### 4.3.2. Singular Spectrum Analysis

**Figure 7:**
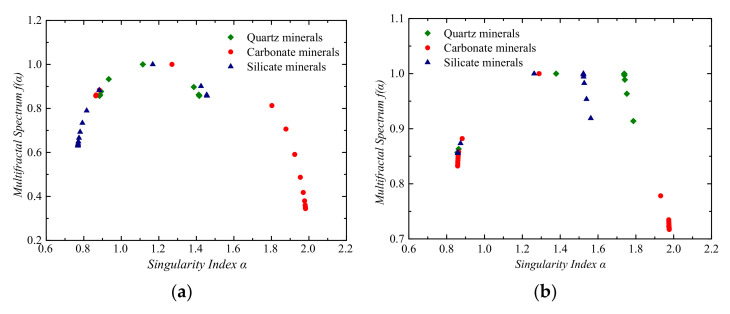
Multifractal singular spectral function. (**a**) Undisturbed loess; (**b**) Lime-treated loess.

The multifractal singular spectrum of PSD of three different minerals in undisturbed loess and lime-treated loess is shown in Figure 7. The α−α(q) functions of PSD of the three mineral particles in the two soils are convex functions, indicating that different mineral particles showed non-uniformity.

Symmetry $\Delta f=f(\alpha_{\min})-f(\alpha_{\max})$ reflects the shape characteristics of multifractal spectrum function. When Δf<0, f(α) was in the form of a right hook; when Δf>0, f(α) was in the form of a left hook [19]. From Figure 7, it can be seen that in undisturbed loess, the Δf of quartz mineral particles equaled 0 with a uniformly symmetrical shape, whereas the carbonate mineral particles Δf>0, f(α) showed a left hook, and feldspar mineral particles Δf<0, f(α) showed a right hook. In the lime-treated loess, quarts and feldspar mineral particles Δf<0, f(α) was in the form of a right hook, whereas carbonate mineral particles Δf>0, f(α) showed a left hook. Except the uniform symmetry of the quartz particles α−α(q) in the undisturbed loess, the other two mineral particles α−α(q) and the quartz, carbonate, and feldspar mineral particles α−α(q) in the lime-treated loess were obviously asymmetric. The more obvious the asymmetry is, the greater the percentage content of the mineral particles changes with the particle size.

According to the multifractal theory, f(α) and D(q) are correlated, and the spectral height of the multifractal spectrum is the maximum value of f(α), namely, the fractal dimension D0 when q=0. The spectral width $\left(\Delta \alpha =\alpha_{\max}-\alpha_{\min}^{}\right)$ can reflect the non-uniformity of probability measure distribution on the whole fractal structure [23].

Takele et al. [25] believed that when Δα is 0, D(q) equals D_0_, remaining unchanged with the increase of q. The larger Δα is, the more uniform PSD is. Its distribution characteristics can be described by multifractal rather than single fractal. Table 1 shows that the spectral widths Δα of quartz, carbonate, and feldspar mineral particles in natural loess were 0.5311, 1.1175, and 0.6883, respectively. The spectral widths Δα of quartz, carbonate, and feldspar mineral particles in lime-reinforced loess were 0.9289, 1.1183, and 0.7026, respectively. It indicates that the non-uniform distribution of carbonate minerals was more obvious than that of quartz and feldspar minerals in these two soils. The greater Δα is, the greater the non-uniform degree of particles is. Therefore, the non-uniformity of different mineral particles in two soils can be obtained as follows: carbonate minerals in lime-treated loess > carbonate minerals in undisturbed loess > quartz minerals in lime-treated loess > feldspar minerals in lime-treated loess > feldspar minerals in undisturbed loess > quartz minerals in undisturbed loess.

## 5. Conclusions

Through image scanning, processing, and mineral composition identification, the obtained multi-scale microstructure images were more representative and could better present the characteristics of macroscopic soil samples. In this work, fractal theory was used to quantitatively study the distribution of mineral particles in undisturbed loess and lime-treated loess. The following main conclusions may be drawn:

1. The skeleton particles of undisturbed loess were obvious and the soil structure was loose, whereas the skeleton particles of lime-treated loess decreased, fine particles were connected with each other, and the soil structure changed from loose to dense. The particle size of each mineral particle in lime-treated loess decreased, and the distribution of carbonate mineral particles was the most non-uniform.
2. Mineral particles in undisturbed loess and lime-treated loess did not conform to the single fractal distribution characteristics, whereas the overall particle distribution had fractal characteristics.
3. The α−α(q) distribution of mineral particles in lime-treated loess was obviously asymmetric, indicating that the percentage of mineral particles in the soil varies greatly with particle size.
4. The non-uniform degree of mineral particles in the two soils was as follows: carbonate minerals in lime-treated loess > carbonate minerals in undisturbed loess > quartz minerals in lime-treated loess > feldspar minerals in lime-treated loess > feldspar minerals in undisturbed loess > quartz minerals in undisturbed loess.

It can be seen that the non-uniformity of mineral particles in lime-treated loess was relatively more obvious than that in undisturbed loess, which is consistent with the observation of multi-scale microscopic images and the results of mineral particle size distribution curves. The analysis in this work can better provide a basis for the future study of the different soil mechanical properties of undisturbed loess and lime-treated loess, and also provide a method for the study of the mechanical properties of different types of soil.

## Figures and Tables

**Table 1:** Multifractal parameters of different mineral particles in undisturbed loess and lime-treated loess.

Multifractal Parameters	Undisturbed Loess			Lime-Treated Loess		
	Quartz Minerals	Carbonate Minerals	Feldspar Minerals	Quartz Minerals	Carbonate Minerals	Feldspar Minerals
D(0)	1	1	1	1	1	1
D(1)	0.9331	0.8832	0.8826	0.8632	0.8821	0.8734
D(2)	0.9072	0.8411	0.8411	0.8585	0.8688	0.8621
$D_{\min}$	0.8872	0.7842	0.7842	0.8578	0.8611	0.8595
$D_{\max}$	1.3645	1.8331	1.4025	1.6714	1.8612	1.4750
ΔD	0.4773	1.0488	0.6183	0.8136	1.0001	0.6155
D(1)/D(0)	0.9331	0.8832	0.8826	0.8632	0.8821	0.8734
Spectral width Δα	0.5311	1.1175	0.6883	0.9289	1.1183	0.7026
Degree of symmetry Δf	0	0.5127	−0.2269	−0.0568	0.1152	−0.0617

## Data Availability

Data sharing is not applicable for this article.
